# A Case of Undiagnosed Sleep Disorder with Hearing Difficulty and Dizziness

**Published:** 2016-03

**Authors:** Fumiyuki Goto, Miki Arai, Mitusru Kitamura, Tomoko Otomo, Ryoto Nagai, Shuujiro Minami, Takanobu Shimada, Tatsuo Matsunaga, Kouichi Tsunoda, Masato Fujii

**Affiliations:** 1 *Department of Otorhinolaryngology, National Hospital Organization, Tokyo Medical Center, Tokyo, Japan. *

**Keywords:** Dizziness, Insomnia, Sleep, Vestibular

## Abstract

**Introduction:**

The aim of this case report was to investigate the relationship between sleep disorders and audio vestibular symptoms.

**Case Report:**

A case of undiagnosed sleep disorder, presenting as a temporary auditory processing difficulty, is presented. The disorder was initially treated as sudden deafness with dizziness. A 23-year-old male patient complained of acute hearing disturbance despite normal results on pure tone audiometry. The patient was initially administered a steroid injection in the hospital. After treatment, his hearing symptoms improved only slightly and he reported balance difficulty with rightward spontaneous nystagmus. Vestibular rehabilitation was performed. We also suspected that his hearing symptom was due to an auditory processing difficulty. Despite steroid treatment and vestibular rehabilitation, neither of his symptoms improved. We subsequently identified the presence of insomnia. He was prescribed zolpidem 5 mg, which slightly improved his symptoms, and referred to a sleep specialist for further examination. Polysomnography was performed, which identified restless leg syndrome and sleep disturbance with delayed sleep phase syndrome. After pharmacological treatment, his sleep disturbance, hearing difficulty, and balance disorder completely resolved.

**Conclusion:**

Sleep disorders may provoke reversible auditory processing difficulties. We should carefully evaluate patients for a potentially undiagnosed sleep disorder, even in patients chiefly complaining of intractable sensory dysfunction such as hearing or balance disturbance**.**

## Introduction

Auditory processing disorder (APD) is a collection of disorders affecting the way the brain processes auditory information and is not currently included in mainstream diagnostic classifications. At our facility, we have examined patients complaining of misunderstanding verbal speech despite having normal pure tone hearing function on pure tone audiometry. These patients may have an auditory processing difficulty, which is similar to APD. Individuals with APD usually have a hearing system with a normal structure and function. However, they cannot process the information they hear in the same way as others do, which leads to difficulties in recognizing and interpreting the sounds composing speech. It is thought that these difficulties arise from dysfunction in the central nervous system (1). Insomnia is a common complaint among patients with intractable dizziness and may be underdiagnosed (2). Recently, insomnia was proposed as a possible trigger for Meniere disease and vestibular dysfunction (3,4). Restless leg syndrome (RLS) is another important sleep disorder, but it is uncommon to examine a RLS patient in the Department of Otolaryngology. In this report, we present a case of undiagnosed sleep disorder with RLS presenting as a reversible auditory processing difficulty with dizziness. 

## Case Report

**Fig 1:**
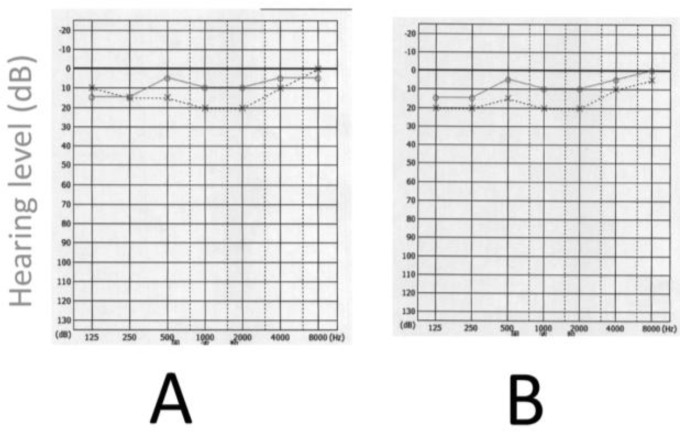
Pure tone audiometry results in a 23-year-old man with acute hearing difficulty. (A) Initial visit; (B) After treatment for restless leg syndrome with pramipexole 0.125 mg

A 23-year-old post-graduate male student was examined for sudden deafness. In May 2014, he was suddenly unable to hear clearly, although he could still hear sound. Pure tone audiometry was performed and was normal, with 7.5d B in the right ear and 16.3d B in the left (Fig. 1A). The results of SDS (speech discrimination score) were normal. Despite this, he was adamant in his complaint and was experiencing difficulty listening in class. The result of his MRI was normal and we could not diagnose any 

organic disorder. The cause of his symptoms

was unclear, but because the symptoms were similar to sudden deafness, he was administered an injectable steroid for 7 days. However, his hearing disturbance improved only slightly and he began to experience balance problems. Repeat examination revealed a spontaneous nystagmus to the right and a bedside head impulse test indicated left-sided canal paralysis. Static posturography showed a slight increase in the total length of path (LNG) and environmental area (ENV.AREA), as shown in (Table. 1). 

**Table 1:** Static posturography performed before and after treatment for RLS and sleep phase disorder

	**before**		**after**	
	Eye open	Eye close	Eye open	Eye close
LNG(cm)	69.9	98.6	53.1	61.7
ENV AREA(cm2)	5.9	3.9	1.5	1.4

RLS: restless leg syndrome, LNG: total length of path

ENV. AREA: environmental area

The Dizziness Handicap Inventory (DHI) and Hospital Anxiety and Depression Scale (HADS) scores were 82 (physical= 24, emotional= 26, function= 32) and 23 (anxiety= 12 and depression= 11). During examination 2 weeks later, no spontaneous, gaze-evoked, or positional nystagmus were found. The Fukuda stepping test was normal and the electronystagmogram with caloric test did not reveal any canal paralysis (5). Vestibular rehabilitation was initiated, but did not improve his subjective dizziness. 

After carefully reviewing his clinical history, we determined that he could hear sound normally, but his ability to listen was decreased. In addition, we found that the patient had a sleep disturbance. Zolpidem 5 mg was prescribed, which improved his sleep difficulty. His dizziness improved gradually, but he still experienced dizziness and hearing difficulty. Further investigation of his sleep disorder was recommended and the patient was referred to a sleep specialist. Polysomnography (PSG) was conducted. The sleep time was 413.5 minutes over a 651-minute recording period. Therefore, the sleep efficacy was 56.6%, which was significantly reduced. The Apnea-hypopnea Index (AHI) was 2.3 times/hour, and the Arousal Index was 12.16 times/hour. Arousal due to apnea-hypopnea and periodic limb movement were each 0.94 times/ hour. PLM index was 4.91 times/h. Collectively, these findings clearly indicated insomnia without apnea or hypopnea. RLS was suspected; therefore, pramipexole 0.125 mg was prescribed. His subjective insomnia, dizziness, and hearing disturbance disappeared within 4 days. The pure tone audiometry results were unchanged (Fig. 1B).

The posturography results improved, as shown in Table 1; however, he still required several hours to fall asleep despite taking zolpidem 5 mg. Delayed sleep phase syndrome was strongly suspected and ramelteon 8 mg was prescribed. His sleep rhythm subsequently improved. 

## Discussion

Sleep deprivation is extremely common in contemporary society and is considered a frequent cause of impaired cognitive performance. Sleep deprivation impairs performance on the Random Gap Detection Test (RGDT) and Staggered Spondaic Word Test (SSWT) (6). When a patient’s adament complaints cannot be clearly explained based on physical symptoms and clinical examination, we often suspect a medically unexplained symptom. This patient was first diagnosed and treated for a medically unexplained symptom. Undiagnosed depression or anxiety is considered a common component of medically unexplained symptoms (7). In this case, the HADS score was 12 in anxiety and 11 in depression. Although, this indicated the existence of mild anxiety and depression, we did not consider an antidepressant or anti-anxiety drug necessary. The patient complained of difficulty hearing despite having normal results on pure tone audiometry; therefore, we suspected that the patient had an auditory processing difficulty similar to APD. He first reported symptoms resembling sudden deafness but later realized that he was able to hear sound but was less able to listen. His symptoms disappeared after treating his sleep disorder and the auditory processing difficulty appeared to be reversible. 

Occasionally, we encounter patients with difficulty hearing speech despite normal pure tone audiometry and speech discrimination ratio results. Some of these patients may have an auditory processing disorder. The present patient initially complained of dizziness with spontaneous nystagmus; therefore, left-sided acute vestibular dysfunction was suspected. However, the vestibular symptoms recovered spontaneously as the caloric test did not detect any canal paralysis. Unsurprisingly, conventional steroid treatment and vestibular rehabilitation did not improve his physical symptoms. 

Reportedly, sleep apnea is closely related to vestibular dysfunction and Meniere disease. However, a clear association between vestibular dysfunction and sleep disorders other than sleep apnea has not been described. We treated the patient’s sleep disorder with zolpidem initially, which slightly improved his subjective dizziness. We then treated his RLS pharmacologically and identified his sleep phase disorder. Treating all of these problems simultaneously completely resolved his hearing and balance problems. It is clear that the sleep disorder played an important role in the onset of his symptoms.

We seldom encounter patients with sleep disorders other than sleep apnea in the Department of Otolaryngology. However, it is important for clinicians to consider a sleep disorder as a cause of hearing and balance problems. Therefore, if the physical symptom does not completely resolve following conventional treatment by otolaryngologists, we should consider a possible sleep disorder.

If a sleep disorder is suspected, it is necessary not only to prescribe appropriate medication, but also to refer the patient for examination by a sleep specialist. It is important to uncover hidden sleeping disorders that may be triggering sensory problems.

## Conclusion

 An undiagnosed sleep disorder is an important cause of MUS and should be considered in cases of auditory processing difficulty and unexplained dizziness. When a sleep disorder is suspected, pharmacological treatment alone is an inadequate solution. Further clinical examination by a sleep specialist may identify an important sleep disorder causing the medically unexplained symptom. 
